# Anetodermic pilomatrixomas: A case series

**DOI:** 10.1002/ski2.284

**Published:** 2023-09-21

**Authors:** Evangelia Vetsiou, Julia Gass, Andre Khoo, Sarah McDonald, Niki Stefanos, Ed Rytina, Nigel Burrows

**Affiliations:** ^1^ Department of Dermatology Addenbrooke's Hospital Cambridge University Hospitals NHS Foundation Trust Cambridge UK; ^2^ Department of Histopathology Addenbrooke's Hospital Cambridge University Hospitals NHS Foundation Trust Cambridge UK

## Abstract

Pilomatrixoma is a benign hair follicle tumour. Anetodermic changes overlying pilomatrixoma are rare. The aim of this study is to evaluate a case series of patients with a clinical diagnosis of anetodermic pilomatrixoma presenting to our Dermatology Department over a 5‐year period. Eight cases were identified. The median age of onset was 21 years. All cases presented on the upper limbs and trunk with a solitary rapidly evolving tumour, tender on palpation. They had an erythematous protuberant appearance with a wrinkled and atrophic surface. Underlying pilomatrixomas were firm measuring 1–5 cm. Simple excision was carried out in seven cases without postoperative complications. In conclusion, anetodermic pilomatrixoma is a rare variant of this tumour, occurring more frequently on the upper body. It presents with identifiable features and should be differentiated from other skin tumours. Surgical removal is usually the gold standard treatment.

## Graphical abstract

Pilomatrixoma is a benign hair follicle tumour. Anetodermic changes overlying pilomatrixoma are rare and its cause is still unknown. The aim of this study is to evaluate a case series of patients with a clinical diagnosis of anetodermic pilomatrixoma presenting to our Dermatology Department over a 5‐year period.

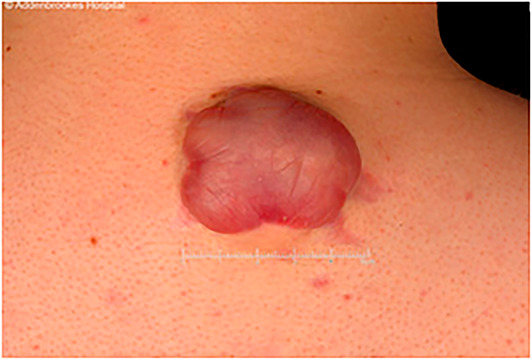

## 1 INTRODUCTION

Pilomatrixoma or calcifying epithelioma, is a benign cutaneous tumour arising from the base of the hair follicle matrix, most commonly occurring in children and young adults and characterised by a solid slow‐growing nodule in the lower dermis which may also extend into the subcutaneous tissue. It is usually located in the head and neck area but may also present on the upper limbs and trunk. Histologically, pilomatrixoma exhibits features of hair follicle matrix cell differentiation. This includes the presence of basal hair matrix cells, transitional cells, eosinophilic amorphous shadow (ghost) cells and calcium deposits in a background of debris.^1^

Anetoderma is a disorder of focal loss of dermal elastic tissue resulting in flaccid, wrinkled skin. Anetodermic pilomatrixoma, also referred to as bullous or pseudobullous pilomatrixoma, is an uncommon variant presenting in only 2% of the cases.^2^ The exact mechanism for its development remains unknown, although trauma has been postulated as a cause.^1^

Multiple pilomatrixomas have been associated with Myotonic Dystrophy type I, Gardner Syndrome, Rubinstein‐Taybi Syndrome, Turner Syndrome, Sarcoidosis and Tuberous Sclerosis.^3^ The aim of this study is to evaluate a case series of patients who presented with anetodermic pilomatrixoma to our Dermatology Department over a 5‐year period.

## 2 REPORT

A retrospective analysis of all cases with a clinical diagnosis of anetodermic pilomatrixoma presenting to our department within the last 5 years was undertaken. Data were collected according the suspected clinical diagnosis, the histopathological features, the treatment and postoperative outcomes.

**FIGURE 1:**
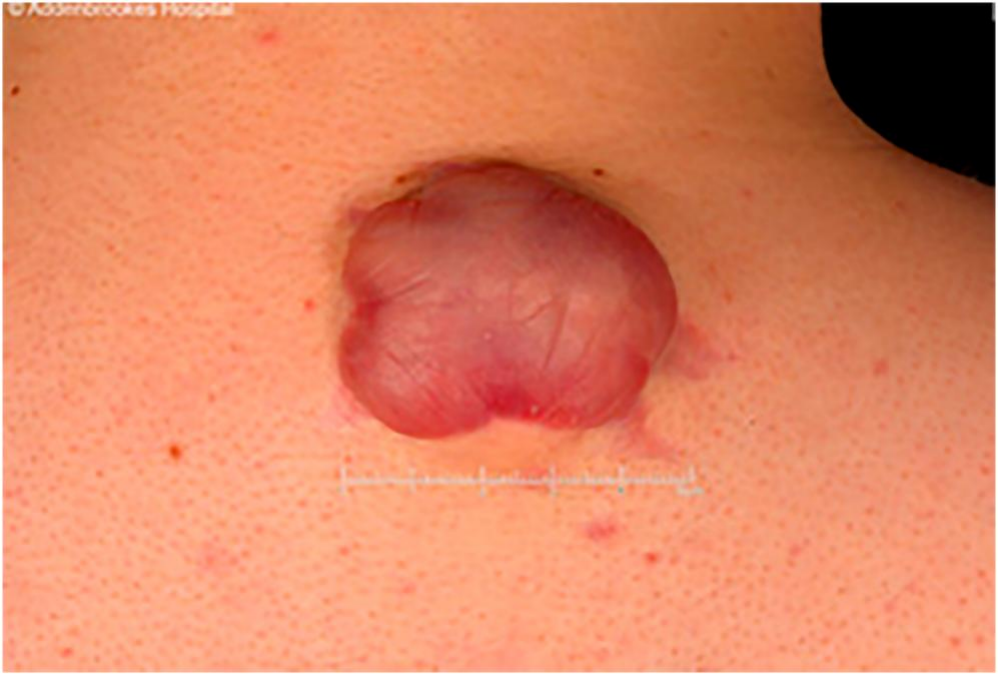
Case 1 showing pink wrinkled balloon like atrophic skin overlying a subcutaneous mass.

**FIGURE 2:**
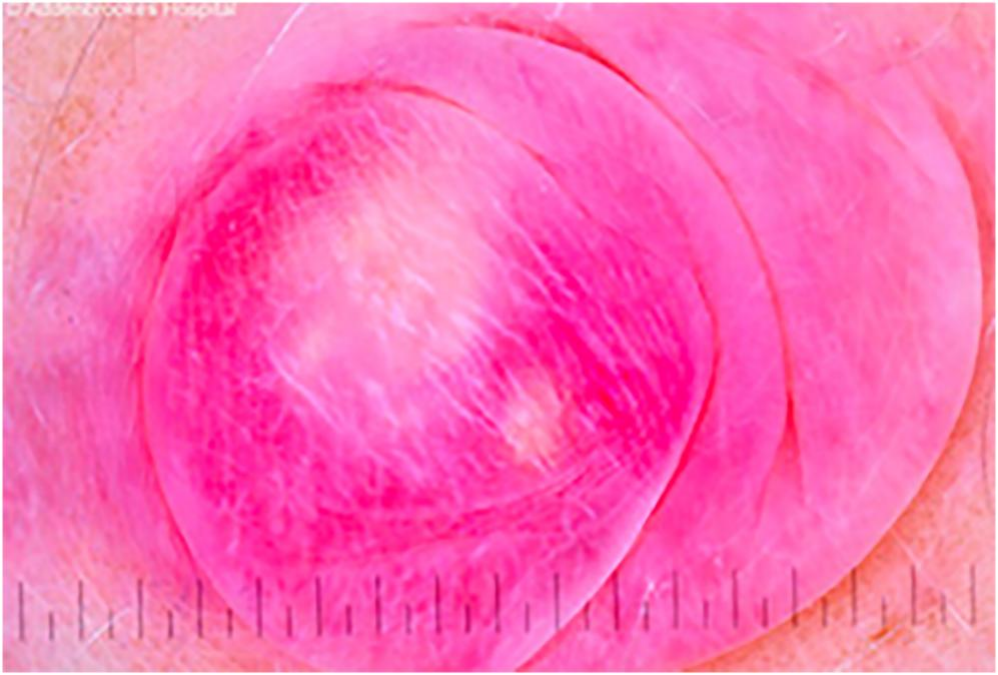
Case 4. Dermoscopy view showing central white structureless area corresponding to firm nodule of pilomatrixoma overlying white streaks on a red background.

Eight cases of anetodermic pilomatrixomas were identified from clinical and pathology records. All were referred as a fast growing tumour. The median age of onset was 21 years. Male to female ratio was 3:1. The patients did not have any comorbidities or joint hypermobility. Clinical suspicion of anetodermic pilomatrixoma (large, atrophic, wrinkled lesion or balloon‐like, atrophic on palpation lesion overlying a firm subcutaneous mass) was made prior to surgical removal (Figures 1 and 2). Dermoscopic examination of the lesions showed irregular linear vessels, white structures, and structureless grayish‐blue areas. The characteristics of the pilomatrixomas are presented in Table 1.

**TABLE 1:** Characteristics of the anetodermic pilomatrixoma cases presented in our Dermatology Department over a 5 year period.

	Case 1	Case 2	Case 3	Case 4	Case 5	Case 6	Case 7	Case 8
	Figure 1			Figure 2	Figure 3			
Gender	Male	Female	Male	Male	Female	Male	Male	Male
Age (years)	19	15	15	20	9	38	13	37
Location	Base of neck	Right shoulder	Back	Left arm	Back	Left thigh	Left shoulder	Right arm
Size of lesion (cm)	5 cm	1.3 × 1.6 cm	1 cm	1.5 × 1.7 cm	4.5 cm	1.5 × 1.5 cm	1 cm	3 × 2 cm
Duration of appearance (months)	6 months	1.5 months	Uncertain	8 months	12 months	2 years	9 years	9 months
Symptoms	Pain	Pain	Asymptomatic	Pain	Pain	Asymptomatic	Pain	Pain
Excision	Yes	Yes	Declined	Yes	Yes	Yes	Yes	Yes

## 3 DISCUSSION

Anetodermic pilomatrixoma is also referred in literature as pseudo‐bullous pilomatrixoma, due to the overlying flaccid skin giving the appearance of a bulla. The diagnosis of this rare subtype is important to exclude the presence of a malignant skin tumour and to be aware of any association with underlying pathologies. Multiple pilomatrixomas have been associated with Myotonic Dystrophy type I, Gardner Syndrome, Rubinstein‐Taybi Syndrome, Turner Syndrome, Sarcoidosis and Tuberous Sclerosis.^3^

The aetiology of anetoderma is uncertain. Secondary anetodermas can develop in association with local infections such as syphilis, tuberculosis and leprosy, inflammatory diseases such as lupus erythematous or tumours^3^ ^,^ ^4^ but these tend to present differently from isolated lesions. It has also been reported that the bulla‐like appearance could result from pinching trauma.^5^ None of our patients had associated systemic abnormalities nor history of trauma.

**FIGURE 3:**
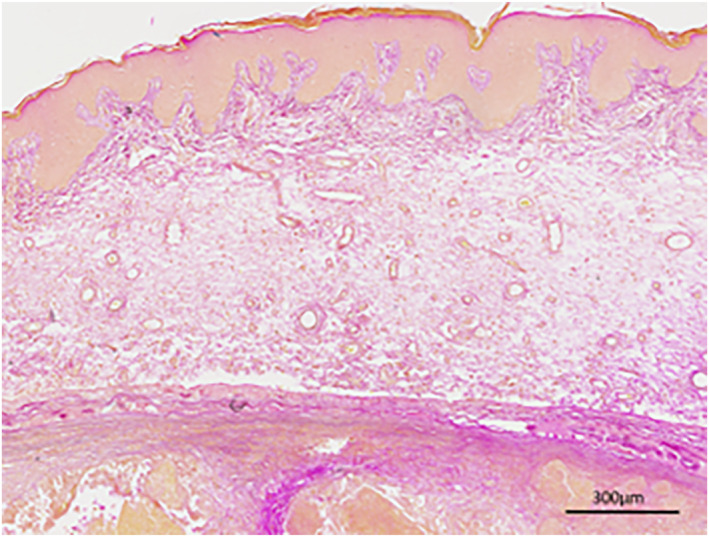
Case 5. At this magnification (40×) an elastic van Gieson stain demonstrates loss of collagen and elastin in the dermis overlying the tumour.

Anetodermic pilomatrixomas have different overlying skin histopathological findings to the common pilomatrixomas. Histology from all our available cases demonstrated dermal atrophy, oedema, mononuclear cell infiltration and lymphatic vascular dilatation, findings in keeping with the diagnosis of anetoderma. It has been suggested that the fluid in the dermis is lymphatic, occurring from lymphatic vessel dilatation beside the tumour (Figure 3).^6^ Hence, induced oedema in the dermis causes continuous mechanical stimulation around the hard pilomatrixoma which leads to lymphatic congestion.^7^ Additionally, it is known that tumour cells produce catabolic enzymes triggered by mechanical stimulation. These enzymes promote the destruction of the collagen and elastic tissue. Whether the recruitment of macrophages to the site of injury releases elastases and collagenases which further escalate the disruption of dermal integrity remains to be further determined. Another hypothesis is that an antecedent disorder, such as infection, inflammatory disorder or tumour predispose the formation of pilomatrixoma that focally inhibits the synthesis of the elastic tissue.^4^ However, further research is warranted for identifying the exact aetiology.

Common pilomatrixomas are located in the head and neck area, while most of the anetodermic variants occur on the upper limbs and trunk where continuous pressure or mechanical irritation is more likely to happen. Our patients presented with rapidly evolving tumours, that were tender on palpation, affecting the upper limbs and back. All of them had erythematous, protuberant appearance with a wrinkled and atrophic surface. Underlying pilomatrixomas were firm measuring 1–5 cm. None of the patients had underlying comorbidities or joint hypermobility. Similar lesions described in the literature vary from 0.5 to 6.0 cm in diameter.^8^ Sonographic findings, such as well defined, non homogenous echogenic nodule with hyperechogenic spots (calcification), are described to facilitate the differentiation as they enable a specific diagnosis; In certain cases, differential diagnosis might include epidermal cysts (hypoechoic masses with no detectable vascularization), haemangiomas (which may or may not contain calcifications) or rare types of skin tumours like dermatofibrosarcoma which usually does not contain calcifications.^2^ No ultrasound imaging was performed in our cases. If required, the treatment of choice is surgical excision, to establish diagnosis, minimise symptoms, such as pain or to improve the appearance due to the increasing size of the lesion. Recurrence is rare.

To our knowledge this is the largest series of anetoderma with pilomatrixomas published in the literature and supports the clinical and histopathological findings by Fujioka et al.^9^ However, larger studies may be required in order to establish the pathogenesis of such tumours.

## CONFLICT OF INTEREST STATEMENT

None to declare.

## AUTHOR CONTRIBUTIONS

**Evangelia Vetsiou**: Data curation (lead); formal analysis (lead); writing – original draft (lead); writing – review & editing (equal). **Julia Gass**: Data curation (supporting); writing – review & editing (equal). **Andre Khoo**: Data curation (supporting); writing – review & editing (supporting). **Sarah McDonald**: Investigation (equal). **Niki Stefanos**: Investigation (equal). **Ed Rytina**: Investigation (lead). **Nigel Burrows**: Conceptualization (lead); supervision (lead); writing – review & editing (equal).

## ETHICS STATEMENT

Patients consented for clinical and photographic data to be used in medical publication.

## Data Availability

The data that support the findings of this study are available on request from the corresponding author. The data are not publicly available due to privacy.
